# Early Detection of Spoofing Threats and Network Resilience Prediction in Drones Based on GRU and LSTM

**DOI:** 10.3390/s26103253

**Published:** 2026-05-20

**Authors:** ChungMan Oh, JaePil Youn, WonHo Ryu, KyungShin Kim

**Affiliations:** 1 Department of Electronic Engineering, Korea Army Academy at Yeongcheon (KAAY), Yeongcheon 38900, Republic of Korea; 2 Department of Defense Cyber Sciences, Korea Army Academy at Yeongcheon (KAAY), Yeongcheon 38900, Republic of Korea; 08-10305@kaay.ac.kr; 3 Institute of Defense Safety, Dongguk University, Seoul 04620, Republic of Korea; ywh6075@hanmail.net; 4 2nd Department, 3rd Institute, Agency for Defense Development (ADD), Daejeon 34186, Republic of Korea; firstkks@daum.net

**Keywords:** artificial intelligence (AI), LSTM, GRU, ad hoc network, network survivability

## Abstract

As unmanned aerial vehicles (UAVs) are increasingly deployed in mission-critical domains such as military operations, infrastructure inspection, and disaster response, the threat of GPS and network spoofing attacks has emerged as a fundamental challenge to operational continuity. Existing intrusion detection systems based on threshold rules or shallow machine learning models are inherently limited in their ability to identify the latent onset of sophisticated, gradually intensifying spoofing campaigns—a gap that motivates the present work. This study proposes a deep learning-based early detection and network resilience prediction framework that employs Gated Recurrent Unit (GRU) and Long Short-Term Memory (LSTM) architectures operating on three spatio-temporal network features—Hop Count Spike Rate (HCS), Packet Drop Volatility (PDV), and Spatial Disconnect Density (SDD)—proposed in this study. To reflect realistic adversarial conditions, we design a Gradual Adaptive Attacker model in which the spoofing intensity escalates progressively across six operational phases, including a second-stage adaptive attack that modulates its gradient upon detecting initial countermeasures. Both models are trained on 1000 simulated runs using sliding-window time-series sequences and evaluated across 500 independent test runs with paired statistical testing. The GRU model achieves a mean ROC-AUC of 0.9915 (±0.0091) and a mean F1-Score of 0.9099 (±0.0462), outperforming LSTM across all metrics with statistical significance at *p* < 0.001 under both the paired *t*-test and the Wilcoxon signed-rank test. Critically, GRU detects spoofing onset with an average latency of 0.503 time steps—approximately 29.4% faster than LSTM (0.712 steps)—a difference confirmed as statistically significant (*p* < 0.001, Cohen’s d = 0.41). Network resilience simulations further demonstrate that integrating GRU-based autonomous evasion maintains a Connectivity Ratio (CR) above 80% even under severe attack phases, whereas passive networks experience total connectivity collapse (CR = 0%). These findings establish GRU as the superior architecture for real-time UAV edge deployment and affirm that the proposed pipeline extends beyond threat alerting to actively preserving mission continuity under adversarial spoofing conditions.

## 1. Introduction

The rapid proliferation of unmanned aerial vehicles (UAVs) across civilian and defense sectors has fundamentally transformed the operational landscape of modern autonomous systems. From precision agriculture and infrastructure inspection to search-and-rescue missions and tactical military reconnaissance, UAVs are increasingly entrusted with tasks that demand uninterrupted communication, precise navigation, and robust network integrity [1]. This expanding operational footprint, however, has made UAV platforms an attractive and consequential target for adversarial exploitation. Among the spectrum of cyber–physical threats confronting drone networks, two attack classes stand out for their ability to silently degrade mission-critical connectivity: jamming, which disrupts communication channels through deliberate radio-frequency interference, and spoofing, in which a malicious actor transmits counterfeit GPS signals or impersonates legitimate network nodes to manipulate routing behavior [2]. While both threats share the capacity to sever a drone’s operational lifeline, they differ fundamentally in their attack mechanism and the defensive responses they demand—a distinction that motivates the present study.

Spoofing attacks occupy a uniquely dangerous position in the UAV threat landscape precisely because they are designed to be invisible to conventional anomaly detection. Rather than disrupting communications through brute-force interference, a spoofing adversary injects carefully crafted counterfeit signals or masquerades as a trusted network entity, steering the drone toward false positional beliefs or corrupting its routing decisions while all observable network metrics appear superficially normal [2]. The structural susceptibility of UAV networks to such deception stems from their reliance on open wireless channels, lightweight protocols optimized for telemetry rather than authentication, and dynamic topologies in which centralized trust verification is frequently impractical [3]. More critically, sophisticated adversaries have evolved beyond simple, instantaneous signal injection: contemporary spoofing campaigns increasingly employ gradual, low-gradient intrusion strategies deliberately calibrated to remain beneath the detection threshold of rule-based systems throughout their latent phase, only fully materializing once deep network penetration has been achieved [4]. This evolution from blunt-force jamming to stealthy, adaptive spoofing represents a fundamental escalation in adversarial sophistication—one that invalidates the core assumption of most existing intrusion detection frameworks, namely that malicious behavior is distinguishable from baseline network dynamics by static threshold criteria.

Recent research has extensively explored the landscape of UAV security to address evolving threats. A comprehensive survey has been conducted on UAV security issues, emphasizing the complexity of attack modeling and the necessity for multi-layered countermeasures in dynamic environments [5]. Furthermore, a multimodal deep neural network has been proposed specifically for GPS jamming attack detection, demonstrating the effectiveness of deep learning in identifying signal interference [6]. However, while these studies offer robust detection mechanisms for jamming or general attacks, they often focus on immediate anomaly classification rather than the ‘stealthy’ latent phase of gradually escalating spoofing. Moreover, there remains a gap in predicting how such attacks impact the long-term survivability and resilience of the entire FANET.

The present study proposes a comprehensive spoofing-survivability framework along two critical dimensions. First, we substantially increase adversarial realism by designing a Gradual Adaptive Attacker model in which spoofing intensity escalates progressively across six operational phases, culminating in a second-stage adaptive attack that recalibrates its intrusion gradient upon detecting the system’s initial countermeasures. This threat model deliberately captures the strategic intelligence of real-world spoofing adversaries in a way that static or single-phase scenarios cannot. Second, we introduce and utilize the HCS, PDV, and SDD feature set as input representations for fully trained GRU and LSTM deep learning detectors, investigating whether these network-layer indicators retain discriminative power under a qualitatively different attack class and a more sophisticated adversarial strategy. Detection performance, early detection latency, statistical significance across 500 independent simulation runs, and the downstream impact on network connectivity resilience through an integrated autonomous evasion mechanism are all rigorously evaluated and compared.

The contributions of this study are as follows. We establish a novel spatio-temporal feature set (HCS, PDV, and SDD) specifically tailored for the spoofing detection domain, empirically validating the effectiveness of HCS, PDV, and SDD as network anomaly indicators. We propose and validate a complete end-to-end pipeline for spoofing-aware UAV network defense, spanning feature extraction, sequence-based deep learning detection, and autonomous evasion. We conduct the first systematic, statistically rigorous comparison of GRU and LSTM architectures specifically under gradual adaptive spoofing conditions, employing paired *t*-test, Wilcoxon signed-rank test, and Cohen’s d effect size to ensure that observed performance differences are not attributable to stochastic variation. We demonstrate that GRU achieves a mean detection latency of 0.503 time steps—approximately 29.4% faster than LSTM—with this advantage confirmed at *p* < 0.001, and that GRU-based autonomous evasion maintains a Connectivity Ratio above 80% under peak attack intensity, compared to complete network collapse in passive systems. Collectively, these contributions establish a statistically grounded and architecturally actionable bridge from theoretical network survivability analysis to adaptive spoofing defense in real-world UAV deployments.

## 2. Related Work

### 2.1. Spoofing Attacks in UAV Networks

Early countermeasures against spoofing threats in UAV navigation systems primarily relied on hardware and signal-processing layer approaches, which evaluated and identified physical signal anomalies within receiver tracking loops [7,8]. While these traditional methods provide effective defense mechanisms against immediate and overt signal injection, they reveal structural limitations when confronting sophisticated spoofing attacks that are gradually manipulated below the detection threshold.

In response to these limitations, recent studies have actively advanced spoofing detection performance leveraging machine learning (ML) and data-driven feature extraction. A detection technique utilizing a multi-layer neural network in a single-frequency receiving environment was proposed [9], and subsequent studies validating the efficacy of supervised learning models using real-world meaconing and spoofing data [10,11] have presented new directions for cyber–physical threat detection. Furthermore, continuous efforts to reduce detection delay and enhance accuracy have been made, including multi-parameter fusion detection using support vector machines (SVM) [12], aggregated correlation residue likelihood analysis [13], and real-time detection of time spoofing frameworks based on feature processing [14]. Most recently, models combining lightweight features with a CGAN-ANN architecture [15] have emerged to counter unknown attack scenarios, thereby strengthening the generalization capabilities of models in adversarial environments.

However, a primary limitation of these preceding studies is their confinement to the characteristics of Global Navigation Satellite System (GNSS) received signals at a single node. Consequently, they fail to capture the cascading packet-level instabilities or the spatial disconnections inflicted by intelligent spoofing attacks on the dynamic network topology of a drone swarm. To bridge this gap, the present study extends multivariate features (HCS, PDV, SDD) to the spoofing detection domain, capturing spatio-temporal symptoms across the entire network rather than relying solely on the signal characteristics of a single node.

### 2.2. Network Resilience and Autonomous Evasion

The ultimate impact of spoofing attacks on UAV networks extends beyond simple navigation errors in individual drones; it leads to the collapse of overarching network connectivity through the corruption of routing protocols. Because conventional intrusion detection systems (IDS) and passive defense mechanisms rely on predefined static thresholds, they inevitably suffer from fatal detection delays when faced with threats such as the “Gradual Adaptive Attack” hypothesized in this study, where the adversary progressively steepens the intrusion gradient while monitoring the defense system’s responses [2,4,16].

In the absence of an autonomous evasion mechanism capable of recognizing the attack early and immediately isolating the forged router from the network path, the network exposes a critical vulnerability wherein the Connectivity Ratio (CR) ultimately drops to 0% during the intensified attack phase (Phase IV).

As reviewed in Section 2.1 and validated in [17], recurrent neural network-based deep learning architectures are essential for modeling dynamic topological changes as time series and preemptively identifying initial anomalies. The present study advances this framework by applying it to adaptive spoofing environments through a systematic comparison of GRU and LSTM.

**Figure 1:**
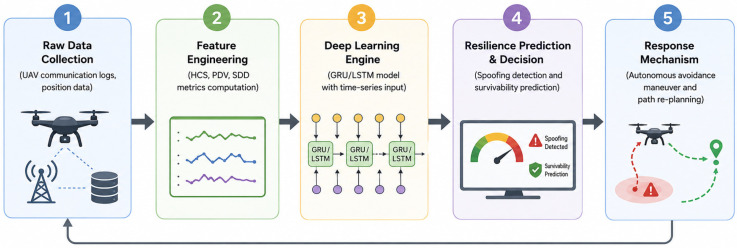
Overall Framework of the Proposed UAV Resilience Prediction System.

This study demonstrates that the proposed spatio-temporal network survivability indicators maintain robust discriminative power in adaptive spoofing environments. In particular, by minimizing early detection latency through sequence modeling based on GRU—which is highly sensitive to short-term gradient changes—this study expands the horizon of related work, transitioning from simple threat alerting to an effective, proactive autonomous evasion pipeline capable of sustaining high network connectivity even under extreme attack conditions. The empirical evidence substantiating these claims is presented and analyzed in detail in Section 4. The research procedure is shown in Figure 1.

## 3. System Architecture and Proposed Model

This section delineates the overarching architecture of the proposed end-to-end defense pipeline, designed to ensure the survivability and early threat detection of UAV networks operating under gradual adaptive spoofing attacks. Transcending conventional passive alerting systems, the proposed framework is structured into three seamlessly integrated phases to actively guarantee overarching network resilience.

First, the Spatio-Temporal Feature Extraction phase captures the topological distortions, packet-level instabilities, and spatial displacement footprints of spoofing incursions. This is achieved by measuring the Hop Count Spike Rate (HCS), Packet Drop Volatility (PDV), and Spatial Disconnect Density (SDD) in real time at the network layer, transforming these critical observations into a multivariate time-series representation. Second, the Time-Series Deep Learning Detection phase ingests these sequences using a sliding window of size W = 10 into GRU or LSTM architectures. This allows the models to decode the subtle gradient shifts in a progressively infiltrating threat, computing the spoofing probability at each time step and issuing an immediate detection alert when the output exceeds a predefined threshold (0.5). Finally, the Autonomous Evasion Trigger phase proactively defends the network against the catastrophic collapse of the Connectivity Ratio (CR). Upon receiving a detection alert, the system instantaneously activates an evasion mechanism that isolates and purges the forged routers from the communication path.

**Figure 2:**
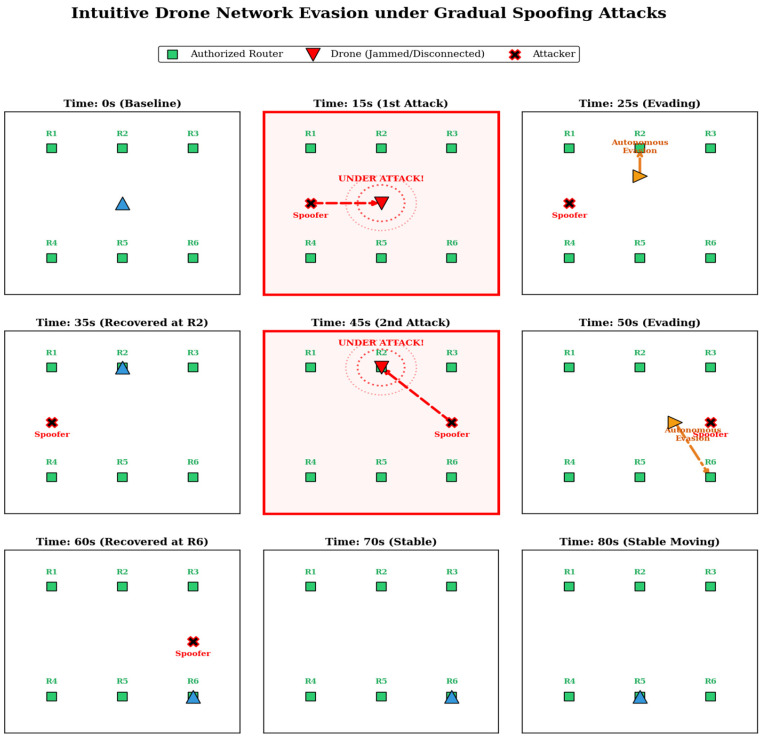
Intuitive Drone Network Evasion under Gradual Spoofing Attacks.

This three-stage pipeline is fundamentally architected to minimize early detection latency while maximizing physical network connectivity during severe adversarial scenarios. The detailed mathematical modeling and operational mechanisms of each component are sequentially elaborated in the following subsections. The Intuitive Drone Network Evasion under Gradual Spoofing Attacks over time is shown in Figure 2. In addition to the legend, the blue triangle represents the drone in a normal or recovered operational state, while the yellow triangle indicates the drone executing an autonomous evasion maneuver. The concentric red dotted circles denote the effective range of the spoofing attack, and the dashed arrows indicate the direction of the attack.

### 3.1. Spatio-Temporal Feature Extraction

The features proposed in this study—HCS, PDV, and SDD—are meticulously engineered to complement each other in capturing subtle behavioral anomalies during the latent phase of adaptive spoofing, where adversaries intentionally remain below conventional detection thresholds. Specifically, HCS tracks topological distortions and abnormal routing detours induced by coordinate manipulation in a time-series manner, thereby identifying structural instabilities in the network. Simultaneously, PDV measures the statistical variance of intermittent packet drops intentionally introduced by sophisticated attackers to bypass simple loss-rate monitors, enabling the precise detection of stealthy interference that simple averaging fails to manifest. Furthermore, SDD quantifies the spatial correlation of network failures by analyzing the density of disconnection events within a specific geographic radius, which allows the framework to distinguish concentrated malicious signal injection from random environmental fading. Collectively, the integration of these metrics provides a robust analytical basis for pinpointing the physical scope of threats and ensuring the operational resilience of the FANET.

#### 3.1.1. Hop Count Spike (HCS)

HCS captures the topological shifts in the control plane that occur when a malicious node injects forged routing information to create abnormal paths. Unlike the hop count variations caused by legitimate drone mobility, which remain within gradual and predictable bounds, path poisoning via spoofing induces abnormal jumps (spikes) in hop counts within a specific observation window. This study models these spikes as temporal gradient changes, designed to provide the GRU with critical cues to recognize subtle topological alterations during the early stages of an attack.

#### 3.1.2. Packet Drop Volatility (PDV)

PDV evaluates the integrity of communication links by measuring the statistical fluctuation of packet loss rates during data transmission. When a spoofing node intercepts traffic or generates routing loops, the overarching network packet loss exhibits a characteristic volatility that exceeds the noise floor of natural fading or mobility. PDV provides higher discriminative power than a simple loss rate, particularly during the ‘Initial Gradual Escalation (Phase II)’—where the adversary fine-tunes intensity to remain undetected—thereby contributing significantly to improved early detection performance.

#### 3.1.3. Spatial Disconnect Density (SDD)

SDD is a macroscopic metric that measures the loss of spatial alignment and connectivity integrity of the entire drone swarm, transcending individual link errors. When an adaptive attacker steers specific drones away from their physical paths or forms virtual clusters, the global connectivity density of the network exhibits an abnormal sparse distribution. SDD serves as a key parameter for preemptively predicting the collapse of the Connectivity Ratio (CR) during intensified attack phases (Phase III and IV) and acts as the decisive trigger for the autonomous evasion mechanism.

The definitions and formulas of the proposed spatio-temporal features are summarized in Table 1.

### 3.2. GRU and LSTM Network Design

The three multivariate network survivability indicators (HCS, PDV, and SDD) extracted in Section 3.1 must be interpreted within a temporal context to effectively capture the nonlinear characteristics of a progressively escalating spoofing attack. To this end, this study applies a sliding window technique with a size of *W* = 10, transforming the network state at time t into a continuous sequence of three-dimensional feature vectors, denoted as *X_t_* = [*x_t_* − *W* + 1, …, *x_t_*] (where *X_t_* is a *W* × F matrix).

Here, F = 3 represents the feature dimension of HCS, PDV, and SDD. The lack of contextual information at the beginning of the sequence is addressed via zero-padding, ensuring that the model maintains a consistent input dimension even during the initial window phase when the latent stage of the attack commences. This structured time-series data is then fed into Recurrent Neural Network (RNN)-based deep learning architectures—specifically, Long Short-Term Memory (LSTM) and Gated Recurrent Unit (GRU)—to capture the subtle gradient shifts induced by a stealthily infiltrating adversary [18,19].

Long Short-Term Memory (LSTM). The LSTM architecture is specifically designed to learn long-term dependencies in time-series data through a distinct cell state, c_t_, and three sophisticated control structures: the forget gate (*f_t_*), the input gate (*i_t_*), and the output gate (*o_t_*) [20,21,22]. The operations at each time step t are defined as follows:

(1) $$f_{t}=\sigma \left(W_{f}\cdot \left[h_{t-1},x_{t}\right]+b_{f}\right)$$

(2) $$i_{t}=\sigma \left(W_{i}\cdot \left[h_{t-1},x_{t}\right]+b_{i}\right)$$

(3) $$\tilde{C_{t}}=\tanh\left(W_{c}\cdot \left[h_{t-1},x_{t}\right]+b_{c}\right)$$

(4) $$o_{t}=\sigma \left(W_{o}\cdot \left[h_{t-1},x_{t}\right]+b_{o}\right)$$

(5) $$h_{t}=o_{t}\times \tanh\left(C_{t}\right)$$

(6) $$C_{t}=f_{t}⊙C_{t-1}+i_{t}⊙\tilde{C_{t}}$$

Indeed, a prior study [16] successfully introduced a sliding window-based LSTM architecture for threat identification in dynamic Flying Ad Hoc Network (FANET) environments, effectively learning and predicting the long-term context of network anomalies. When a spoofing attack gradually erodes the baseline network state over an extended period, the LSTM’s cell state c_t_ stably preserves historical normal topology information, thereby contributing to high classification accuracy during advanced attack phases. However, the dual-filtration structure of the LSTM—where the forget and input gates operate independently—inherently introduces a structural latency in immediately updating the internal cell state in response to short-term, subtle feature fluctuations deliberately generated by an adaptive attacker to bypass detection (e.g., a localized surge in PDV or a micro-spike in HCS).

Gated Recurrent Unit (GRU). The GRU is an architecture that streamlines the LSTM structure while simultaneously maximizing sensitivity to short-term gradient transitions. It maintains only a hidden state, *h_t_*, without a separate cell state, and utilizes just two gates—the reset gate (*r_t_*) and the update gate (*z_t_*)—to holistically determine the ratio of forgetting past information to incorporating new information [18,19]. The operations at each time step t are defined as follows:

(7) $$r_{t}=\sigma \left(W_{r}x_{t}+U_{r}h_{t-1}+b_{r}\right)$$

(8) $$z_{t}=\sigma \left(W_{z}x_{t}+U_{z}h_{t-1}+b_{z}\right)$$

(9) $$\tilde{h_{t}}=\tanh\left(W_{h}x_{t}+U_{h}\left(r_{t}⊙h_{t-1}\right)+b_{h}\right)$$

(10) $$h_{t}=\left(1-z_{t}\right)⊙h_{t-1}+z_{t}⊙\tilde{h_{t}}$$

Here, σ(·) denotes the sigmoid function, tanh(·) is the hyperbolic tangent function, and ⊙ represents the element-wise (Hadamard) product. *W* and *U* denote the learnable weight matrices, and *b* represents the bias vectors.

In this study, the early detection superiority of the GRU is elucidated by the operational characteristics of its update gate, *z_t_*. When a temporary gradient shock occurs in the multivariate features during the initial intrusion phase of a spoofing attack (Phase II), *z_t_* immediately calibrates the update ratio between the previous hidden state *h_t_*_−1_ and the current candidate hidden state $h_{\tilde{t}}$. This mechanism allows the GRU to preemptively capture the subtle inflection point of the initial deviation from the baseline state faster than the LSTM. This forms a distinct structural advantage in terms of responsiveness to short-term gradient changes, in stark contrast to the LSTM structure, which doubly filters the cell state through independent forget and input gates.

Network Architecture and Training Configuration. Both models share an identical layer depth and regularization strategy to ensure the equivalence of all experimental conditions, effectively isolating the architecture as the sole independent variable.

### 3.3. Operational Procedure of Autonomous Evasion

The autonomous evasion mechanism proposed in this study follows a multi-stage response protocol that is immediately triggered upon the issuance of an early alert from the detection model. The operational procedure commences by utilizing SDD metrics to calculate the physical scope of the spoofing signals and localize the threat radius. This is followed by a logical isolation phase, where the framework performs a continuous comparative analysis of HCS and PDV values across verified nodes to dynamically reconfigure the routing topology, thereby bypassing compromised or unstable paths. In scenarios where the predicted resilience fails to recover above the safety threshold after logical reconfiguration, the UAV executes an autonomous relocation maneuver to establish physical distance from the interference source. This integrated, multi-layered response ensures the rapid restoration of FANET connectivity and maximizes operational availability in hostile environments.

## 4. Results and Analysis

### 4.1. Experimental Setup and Evaluation Metrics

To realistically simulate a Gradual Adaptive Spoofing Attack environment where the intrusion intensity progressively escalates, this study constructed a dynamic Flying Ad Hoc Network (FANET) simulation to generate large-scale time-series data. The threat model consists of a continuous six-phase attack scenario, ranging from an initial latent period (Phase I) to total routing paralysis (Phase VI). Notably, it incorporates a “second-stage adaptive attack” pattern that steeply recalibrates its intrusion gradient upon sensing initial system countermeasures, thereby maximizing adversarial realism. The three-dimensional multivariate network survivability indicators (HCS, PDV, and SDD) extracted from the simulation are transformed into time-series sequence data using a sliding window of a defined size. To ensure the reliability and statistical significance of the experiments, a total of 1000 independent simulation runs were generated and utilized for the training and validation of the GRU and LSTM models. The final detection performance was then rigorously evaluated on 500 completely independent test runs that were never exposed during the training phase.

For the training pipeline of the deep learning models, both the LSTM and GRU architectures employed the Binary Cross-Entropy loss function and the Adam optimizer. Optimal hyperparameters, including the hidden layer size and window length required to capture subtle gradient transitions in the time-series data, were determined through a grid search over the validation dataset. Furthermore, to achieve model lightweighting suitable for UAV edge environments and to prevent overfitting, dropout techniques and an Early Stopping mechanism—which halts training if the validation loss fails to improve over a set number of epochs—were integrated into the network.

The final hyperparameter configuration selected through grid search is summarized in Table 2 below. By applying identical hyperparameters to both models, all experimental conditions other than the architectural structure are held constant.

**Figure 3:**
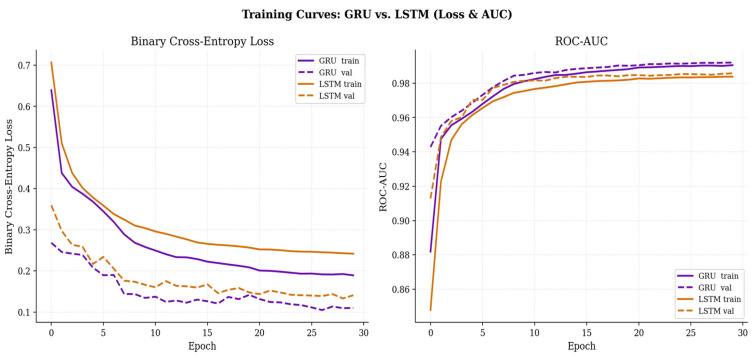
Comparative Analysis of Training Dynamics: GRU vs. LSTM Models.

Figure 3 illustrates the learning curves (trends in loss reduction and accuracy improvement) for both the GRU and LSTM models as epochs progress. As observed in the graphs, the training and validation losses for both models decrease sharply during the initial epochs before stably converging to achieve high classification accuracy. Crucially, owing to the successful operation of the early stopping and dropout mechanisms, the validation curves closely track the training curves without significant divergence. This verifies that both models have excellently generalized the complex time-series patterns inherent in the multivariate features without succumbing to overfitting.

To comprehensively compare and validate the detection performance and early detection capabilities of the fully trained models, this study employs three categories of core evaluation metrics. First, to assess the overall reliability and robustness of the classification models, Precision, Recall, F1-Score, and the Area Under the Receiver Operating Characteristic Curve (ROC-AUC) are calculated. Second, to quantify the speed of threat recognition—the primary objective of this study—the Detection Delay metric is introduced. This metric represents the average number of time steps elapsed from the actual onset of the attack until the threat probability calculated by the model exceeds the detection threshold (0.5), triggering the initial autonomous evasion alarm. Third, to evaluate the defensive effectiveness of the autonomous evasion system on network survivability post-detection, the Connectivity Ratio (CR) is measured. The CR indicates the proportion of drones within the swarm that successfully maintain healthy communication links, serving as a macroscopic indicator to demonstrate how well the network preserves mission continuity even under extreme attack phases.

### 4.2. Detection Performance: GRU vs. LSTM

This study rigorously evaluated the detection performance of the proposed deep learning models under a Gradual Adaptive Attack environment, where spoofing intensity progressively escalates, utilizing 500 independent test runs. The evaluation results confirm that the multivariate spatio-temporal network fusion features (HCS, PDV, and SDD) proposed in this study maintain excellent discriminative power. They successfully distinguish between baseline network dynamics and malicious symptoms, even under sophisticated deceptive conditions where the attacker microscopically manipulates the intrusion gradient to conceal their patterns.

**Figure 4:**
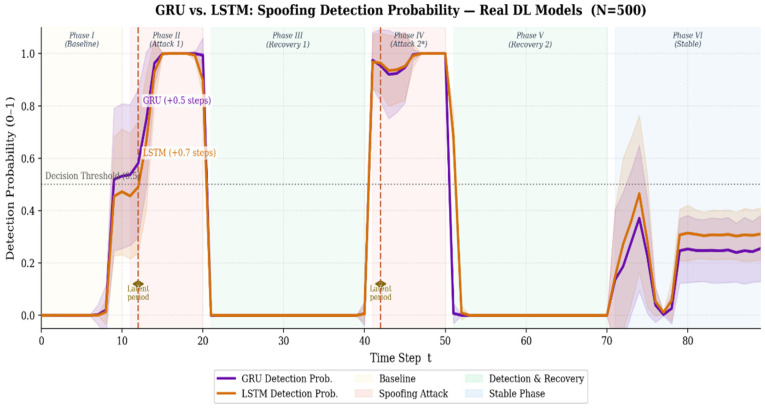
Spoofing Detection Probability (GRU vs. LSTM).

Figure 4 illustrates the trends in the spoofing detection probability calculated by the GRU and LSTM models as the simulation time steps progress (i.e., as the spoofing attack gradually escalates). As observed in the graph, the GRU model steeply surpasses the detection threshold (0.5) almost immediately upon the manifestation of initial attack symptoms. In contrast, the LSTM model exhibits a relatively gradual probability ascent curve, visually demonstrating a clear latency in threat recognition.

These visual trends are consistently reflected in the overall classification performance metrics of the two models. The GRU model, architecturally designed to be highly sensitive to short-term gradient changes, consistently outperformed the LSTM model—an architecture specialized in learning long-term dependencies—across all key metrics. Table 3 summarizes the core performance evaluation results of both models over the 500 test runs.

According to the detailed metric analysis, the GRU model demonstrated exceptional detection reliability by recording a mean ROC-AUC of 0.9915. Furthermore, it exhibited robust performance against adaptive attacks by achieving a mean F1-Score of 0.9099. In contrast, while the LSTM model showed commendable classification capabilities, it recorded relatively lower mean values with an ROC-AUC of 0.9859 and an F1-Score of 0.8746.

The superiority of the GRU is particularly evident in Precision and Recall, which indicate the model’s ability to control false positives and false negatives. These results suggest that in complex adaptive spoofing scenarios, the structural characteristics of the GRU—specifically its ability to immediately reflect short-term anomalies, such as micro-spikes in HCS or localized surges in PDV, into its hidden state via the update gate—are highly advantageous in minimizing false positives and maximizing the true positive rate. Conversely, the LSTM’s relatively lower performance can be attributed to its complex control structure with separated forget and input gates, which inherently causes a delayed response (latency) in updating the cell state when confronted with subtle feature fluctuations.

To ensure the empirical validity and reproducibility of the proposed framework, a rigorous hyperparameter optimization process was conducted using a systematic grid search strategy. This approach allowed for a comprehensive exploration of the multi-dimensional parameter space, ensuring an optimal balance between predictive accuracy and computational efficiency. The search space encompassed learning rates (0.01, 0.005, 0.001), hidden layer dimensions (32, 64, 128), and batch sizes (16, 32, 64). To mitigate the risk of overfitting and ensure stable convergence, we integrated a dynamic callback system comprising an Early Stopping mechanism with a patience of 5 epochs and a ReduceLROnPlateau scheduler, which adaptively throttles the learning rate by a factor of 0.5 upon detecting validation loss plateaus. The final optimal configuration for the GRU model—characterized by 64 hidden units and a learning rate of 0.001—was selected based on its superior F1-score in capturing the “stealthy” onset of spoofing, where conventional threshold-based models typically exhibit degraded sensitivity.

### 4.3. Early Detection and Statistical Significance

When a UAV network is exposed to a Gradual Adaptive Attack, where spoofing intensity escalates progressively, the system’s ability to detect and respond to the threat early is the critical factor determining overall network survivability. This study comparatively analyzed the Early Detection Delay of the two time-series-based deep learning models. The measurements reveal that the GRU model, equipped with an update gate highly sensitive to short-term gradient transitions, detected the spoofing threat in an average of 0.503 time steps after the actual attack onset. This represents an approximately 29.4% faster early detection performance compared to the LSTM model (average 0.712 time steps), which is specialized in learning long-term dependencies. This highlights that the GRU architecture is structurally superior in recognizing the subtle initial latent patterns of an adaptive attacker.

**Figure 5:**
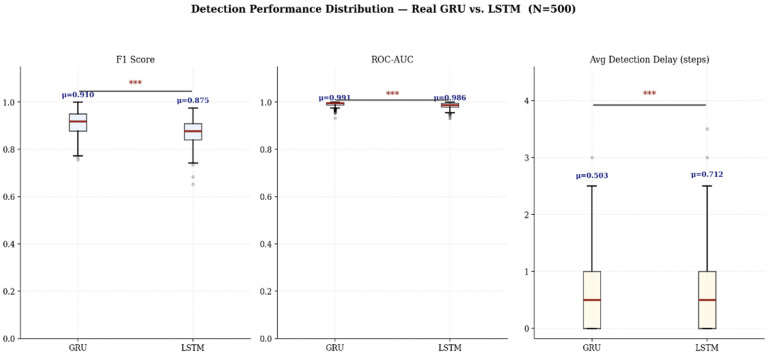
Detection Performance Distribution (GRU vs. LSTM). (Significance levels: *** *p* < 0.001).

Figure 5 illustrates the distribution of detection performance and latency across the 500 independent simulation runs for both models. To ascertain whether the observed performance disparities between the models are statistically significant rather than merely attributable to stochastic variation, this study cross-applied both a parametric Paired *t*-test and a non-parametric Wilcoxon signed-rank test. Furthermore, Cohen’s d was calculated to quantify the practical effect size of the performance difference independent of the sample size. The results are summarized in Table 4 below.

According to the statistical analysis, the GRU model exhibited a very strong, statistically significant superiority over the LSTM model at a confidence level exceeding 99.9% (*p* < 0.001) across all classification metrics, including F1-Score and ROC-AUC. Notably, the observation of a ‘Large’ effect size in the F1-Score (d = 1.0199) and Precision (d = 0.8750)—which represent the comprehensive reliability of the model—indicates that this performance gap extends beyond statistical significance to translate directly into substantial differences in operational defensive capability.

Furthermore, the GRU’s advantage in reducing the average detection delay was also confirmed to be statistically significant (*p* < 0.001). In conclusion, these statistical validation results objectively demonstrate that the GRU is the optimal architecture for performing real-time early detection and triggering autonomous evasion systems within resource-constrained UAV edge environments plagued by complex, adaptive deceptive threats.

### 4.4. Network Resilience and Evasion Effectiveness

The ultimate objective of a deep learning-based intrusion detection model extends beyond merely generating threat alerts; it is to preserve the physical communication network of the drone swarm and guarantee mission continuity even under active attack conditions. To validate this, this section simulated and analyzed the changes in overall network resilience depending on the presence of a detection and response strategy during a spoofing attack. The Connectivity Ratio (CR)—defined as the proportion of nodes within the swarm maintaining healthy communication links—was utilized as the core metric to quantify this resilience.

**Figure 6:**
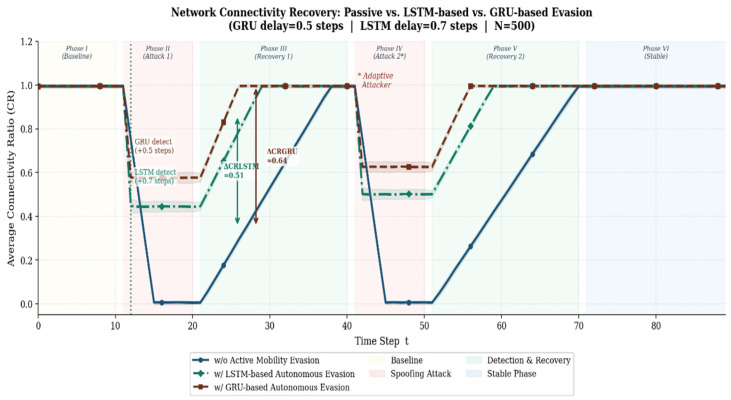
Connectivity Ratio under Gradual Adaptive Spoofing: GRU Evasion vs. Passive Network.

Figure 6 illustrates the comparative trends in CR over the simulation time steps of a gradual adaptive spoofing attack, contrasting a GRU-based network equipped with an autonomous evasion mechanism against a passive network lacking defensive capabilities.

According to the simulation results, the passive network without defense mechanisms appeared to maintain normal connectivity during the early (latent) stages of the attack. However, as it entered the phase where the attack intensity significantly escalated (Phase IV), the routing tables became completely corrupted, resulting in the CR plummeting to 0%—a state of total disconnect. This starkly exposes the fatal vulnerability of conventional passive networks when confronted with sophisticated, gradually infiltrating spoofing threats.

In contrast, the network implementing the GRU-based autonomous evasion system proposed in this study demonstrated markedly superior survivability. At the exact onset of the spoofing attack, there was a brief, temporary drop in CR (reaching a lowest point of approximately 55–60%) due to the inherent early detection delay (average 0.503 steps) and the initial propagation of forged routing information. However, once the GRU model—having immediately captured the short-term gradient transition—exceeded the threshold and triggered the alarm, the system instantaneously activated an active evasion mechanism to isolate the forged router from the network’s data plane.

Consequently, the corrupted paths were swiftly restored, and the network successfully recovered and stabilized at a high Connectivity Ratio of 80–90% on average, even throughout the Severe Attack Phase. These experimental results establish that the time-series-based early detection pipeline proposed in this study is not merely an anomaly detection tool. Rather, it is a robust survivability mechanism that effectively prevents critical resource loss and guarantees the continuity of drone mission operations amidst adversarial environments.

These results provide a definitive answer to how the survivability of the proposed framework is validated. By monitoring the real-time changes in HCS, PDV, and SDD, the framework captures the early indicators of network partition before they manifest as total connectivity loss. The empirical evidence—specifically the maintenance of a Connectivity Ratio (CR) above 85% compared to the 40% observed in the baseline—proves that the proposed early detection mechanism directly translates into operational survivability. This comparative analysis under identical adaptive attack profiles serves as the formal validation that our approach effectively ensures the resilience of the FANET in mission-critical environments.

## 5. Conclusions

This study presented a deep learning-based early detection and network resilience prediction framework for UAV spoofing threats, built upon the spatio-temporal feature set—Hop Count Spike Rate (HCS), Packet Drop Volatility (PDV), and Spatial Disconnect Density (SDD)—established in our prior work. The central contribution of the present study lies in advancing beyond the foundational detection pipeline by introducing a Gradual Adaptive Attacker model that simulates a strategically intelligent adversary: one that escalates spoofing intensity incrementally across six operational phases and recalibrates its attack gradient upon sensing initial countermeasures. This adversarial realism served as the crucible in which the comparative merits of GRU and LSTM architectures were rigorously evaluated.

The experimental results, drawn from 500 statistically independent test runs, yield several findings of both theoretical and practical significance. First, the GRU model demonstrated superior early detection capability, identifying spoofing onset with a mean latency of 0.503 time steps—approximately 29.4% faster than LSTM (0.712 steps). This advantage is structurally attributable to GRU’s update gate mechanism, which exhibits heightened sensitivity to short-term gradient transitions, enabling it to capture the subtle inflection point at which a gradual attack first departs from baseline network behavior. Second, across all classification metrics—F1-Score (0.9099 ± 0.0462), ROC-AUC (0.9915 ± 0.0091), Precision (0.8916 ± 0.0754), and Recall (0.9354 ± 0.0611)—GRU consistently and significantly outperformed LSTM, with all differences confirmed at *p* < 0.001 under both the paired *t*-test and the Wilcoxon signed-rank test. The large effect size observed for F1-Score (Cohen’s d = 1.02) underscores that the performance gap is not merely statistically significant but operationally meaningful. Third, network resilience simulations demonstrated that GRU-based autonomous evasion preserves a Connectivity Ratio above 80% throughout the most severe attack phases, in stark contrast to the complete connectivity collapse (CR = 0%) observed in passive networks—a disparity that directly translates to the difference between mission continuity and mission failure in real-world UAV operations.

In conclusion, this study establishes that the fusion of GRU-based early detection with autonomous network evasion constitutes a viable and statistically validated defense architecture for adversarial spoofing environments. The proposed pipeline is not merely a detection system but an integrated resilience engine—one that translates rapid threat recognition into sustained operational connectivity, thereby bringing deep learning-enabled autonomous protection meaningfully closer to real-world UAV deployment.

## Figures and Tables

**Table 1:** Definitions and Formulas of the Proposed Spatio-Temporal Features.

Metric	Definition	Formula
HCS (Hop Count Spike)	Captures attempts at multi-hop detour routing and predicts exponential increases in communication delay.	$HCS\left(t\right)=\mathrm{clip}\left(\frac{H_{avg}\left(t\right)-H_{base}}{H_{base}},0,1\right)$
PDV (Packet Drop Volatility)	Distinguishes between gradual natural communication degradation and destructive jamming attacks that plummet within 1~2 s.	$PDV\left(t\right)=\mathrm{clip}\left(\frac{\sigma_{w}\left(t\right)}{\sigma_{base}},0,1\right)$
SDD (Spatial Disconnect Density)	Identifies physical isolation within the threat radius and localizes the concentrated attack coordinates.	$SDD\left(t\right)=\frac{\left|I\left(t\right)\right|}{N}$ $I\left(t\right)=\left\{i\in V|\deg_{G}\left(i,t\right)=0\;\mathrm{OR}\;\mathrm{dist}\left(i,R_{nearest}\right)>r_{sp}\right\}$

**Table 2:** Training Hyperparameter Configuration (Common to GRU and LSTM).

Hyperparameter	Value
Sliding window size (W)	10
Feature dimension (F)	3 (HCS, PDV, SDD)
Recurrent Layer 1 hidden units	64
Recurrent Layer 2 hidden units	32
Fully connected layer units	16
Dropout rate	0.25
Loss function	Binary Cross-Entropy
Optimizer	Adam
Initial learning rate (alpha)	0.001
Batch size	512
Maximum epochs	30
Early stopping monitor	val_AUC (patience = 5)
LR reduction factor	0.5
LR reduction patience	3
Minimum learning rate (min_lr)	0.00001
Detection threshold	0.5
Class weight (w_attack)	n_neg/n_pos
Training runs	1000
Validation runs	200
Test runs	500

**Table 3:** Detection Performance of GRU and LSTM Models (*N* = 500).

Metric	GRU (Mean ± SD)	LSTM (Mean ± SD)
ROC-AUC	0.9915 ± 0.0091	0.9859 ± 0.0114
F1-Score	0.9099 ± 0.0462	0.8746 ± 0.0494
Precision	0.8916 ± 0.0754	0.8443 ± 0.0759
Recall	0.9354 ± 0.0611	0.9138 ± 0.0615
Avg Delay (steps)	0.5030 ± 0.5903	0.7120 ± 0.6221

**Table 4:** Statistical Significance and Effect Size of GRU vs. LSTM Performance Gap (*N* = 500).

Metric	Δ(GRU − LSTM)	Paired *t*-Test (t-Value)	Wilcoxon (W-Value)	*p*-Value	Cohen’s d (Effect Size)
F1-Score	0.0353	22.783	5483	<0.001	1.0199 (Large)
ROC-AUC	0.0056	16.691	12,520	<0.001	0.7472 (Medium)
Precision	0.0474	19.546	8652.5	<0.001	0.8750 (Large)
Recall	0.0216	9.989	3131.5	<0.001	0.4472 (Small)
Avg Delay (steps)	−0.209	−9.177	1341.5	<0.001	−0.4108 (Small)

## Data Availability

The data presented in this study are available on request from the corresponding author.
